# Investigation of Psychometric Properties and Correlation with Psychological Distress after Hurricane Hazards in Puerto Rico

**DOI:** 10.3390/ijerph21101267

**Published:** 2024-09-24

**Authors:** Ruthmarie Hernández-Torres, Mary Rodríguez-Rabassa, Lianel P. Rosario, Cristina Peña-Vargas, Zindie Rodríguez-Castro, Idhaliz Flores, Francisco Cartujano-Barrera, Rosario Costas-Muñíz, Nelmit Tollinchi-Natali, Estefania Torres-Marrero, Ernesto Rosario-Hernández, Heather Jim, Guillermo N. Armaiz-Pena, Eida M. Castro-Figueroa

**Affiliations:** 1 Department of Psychiatry and Behavioral Sciences, Memorial Sloan Kettering Cancer Center, New York, NY 10065, USA; costasmr@mskcc.org; 2 School of Behavioral & Brain Sciences, Ponce Health Sciences University, Ponce 00716, Puerto Rico; marodriguez@psm.edu (M.R.-R.); lrosario21@stu.psm.edu (L.P.R.); cpena@psm.edu (C.P.-V.); ntollinchi@psm.edu (N.T.-N.); etorres17@stu.psm.edu (E.T.-M.); erosario@psm.edu (E.R.-H.); ecastro@psm.edu (E.M.C.-F.); 3 Ponce Research Institute, Ponce Health Sciences University, Ponce 00716, Puerto Rico; zrodriguez@psm.edu (Z.R.-C.); iflores@psm.edu (I.F.); 4 School of Medicine, Ponce Health Sciences University, Ponce 00716, Puerto Rico; garmaiz@psm.edu; 5 Department of Public Health, University of Rochester, Rochester, NY 14627, USA; francisco_cartujano@urmc.rochester.edu; 6 Department of Health Outcomes and Behavior, Moffitt Cancer Center, Tampa, FL 33612, USA; heather.jim@moffitt.org

**Keywords:** natural disasters, hurricanes, psychological distress, cancer, psychometric

## Abstract

Background: Puerto Rico (PR) is highly vulnerable to hurricanes, which severely impact cancer survivors by causing healthcare disruptions and increasing stress. This study investigates the reliability and factor structure of the Hurricane Hazards Inventory (HHI) and its relationship with psychological distress among cancer survivors and non-cancer controls in PR. Methods: Using secondary data from a longitudinal study following Hurricane Maria (HM), the baseline assessment included sociodemographic data from participants, HHI, Patient Health Questionnaire (PHQ-8), and Generalized Anxiety Disorder (GAD-7). Statistical analyses involved descriptive statistics, Exploratory Factor Analysis (EFA), and Partial Least Squares Structural Equation Modeling (PLS-SEM). Results: Among 260 participants, 78.7% were women, with a median age of 58.0 years. EFA reduced the HHI to 17 items grouped into three factors explaining 62.6% of the variance with excellent reliability (Cronbach’s alpha 0.91). The three factors also showed good to excellent reliability (alpha 0.81 to 0.92). The median HHI score was 11.0 (range 4.0–26.5) out of 68. PLS-SEM revealed a direct effect of being a cancer survivor and tertiary hazards on depression and anxiety. Conclusion: The HHI is a valid and reliable tool for assessing mental health impact in cancer survivors after hurricanes. However, the study had limitations, including its small sample size and lack of control for all confounding variables. Future research with larger and more diverse samples is needed to further validate the HHI and examine its generalizability.

## 1. Introduction

Puerto Rico is an archipelago in the Caribbean Sea and a non-incorporated territory of the United States (US). The archipelago is susceptible to weather events due to its location and climate. In addition, due to global warming and climate change, tropical storms and hurricanes have become more intense in the past 20 years [1,2]. Climate change has caused extreme weather events, rising sea levels, and warmer oceans. These result in coastal flooding, stronger storms, heavier rains, and faster winds in the event of natural disasters [1,2,3,4].

During the last decade, Puerto Rico has suffered an economic decline, causing a deterioration in water and electric infrastructure [1,5,6]. In the same way, the general population’s social and health services have been affected. In this scenario, in September 2017, Puerto Rico was impacted by two major natural disasters, Hurricane Irma and Hurricane Maria, leaving approximately USD 90 million in damages [7,8]. Hurricane Maria was the worst natural disaster in the past 20 years of Puerto Rico’s history; it was the deadliest of 2017’s three major storms, with over 4000 fatalities [8,9]. The mortality rate increased until December 2017, and one-third of the deaths were attributed to delayed or interrupted healthcare services [10,11]. The hurricane resulted in complete loss of electricity, infrastructure damage, and shortages of food, water, and telecommunications for the entire population [7,12].

Despite Hurricane Maria’s impact documentation, there is limited information on standardized measures to evaluate hurricane hazards and their effect on vulnerable populations such as cancer patients. Little was found regarding multi-hazard instruments evaluating Spanish-speaking populations after a hurricane. The development, validation, and use of tools to measure the impact of hurricane hazards in this population, mainly in chronic illness patients after a natural disaster, are essential to plan and apply interventions designed to improve quality of life and to reduce possible psychological distress and barriers in treatment access [13,14,15].

Various global organizations, including the United Nations, have developed guidelines to manage the aftermath of natural disasters, including hurricanes, earthquakes, tsunamis, landslides, floods, biological disasters, wildfires, coastal disasters, sea-level rises, and tech hazards [16,17,18]. The United Nations has also developed a Handbook for Disaster Assessment focused on strategy formulation to recover after the impact of natural disasters [19]. Additional measurements focus on disaster-related damages by examining the number of fatalities, injuries, and financial damages, considering regions, countries with various levels of wealth and development, and policies [20]. Assessing natural disaster hazards is a complex process, considering the physical impact on the community’s hazard mitigation and emergency preparedness practices [21,22]. Moreover, the availability of resources for these assessments may be limited at both the administrative and research levels [23]. A measure of psychological distress after Hurricane Maria, the Post-Hurricane Distress Scale (PHDS), has been validated in our population. However, it does not separately assess the impact of the disaster in terms of material and social losses [24]. Given this limitation, there is a need for robust instruments that allow the characterization of risks and the description of the multi-hazard effects of a natural disaster [25,26,27,28,29].

Drawing from extensive research on hurricane hazards and experiences with Hurricane Maria, we propose a multi-tiered classification system to categorize the impacts of these events [19,20,30,31,32,33]. This framework is divided into three levels: primary, secondary, and tertiary. Our inspiration for this classification stems from the Hurricane Hazard Inventory (HHI), a tool developed by our research team in the aftermath of Hurricane Maria. The HHI, designed to assess the overall consequences of natural disasters, aims to offer a more granular analysis of hurricane impacts. Primary hazards include high winds, flooding, rain, and heavy waves; secondary hazards involve the loss of electric power, water, food, and other resources; and tertiary hazards result from secondary hazards, affecting aspects such as the loss of loved ones, family support, and family security [19,20,30,31]. These outcomes illustrate the hazards of natural disasters, defined by their location, intensity, and likelihood [32,33,34].

Particularly, secondary and tertiary hazards, like the loss of social support and individual property, significantly affect and reduce the psychological well-being of those who experience them [35]. Moreover, they are associated with psychological symptoms and significant stress levels in the general population [36,37]. Additionally, certain population subgroups, like medically fragile individuals such as cancer survivors, are more vulnerable to the impacts of natural disasters [38,39,40]. An exploratory study involving breast and colorectal cancer patients diagnosed six months after Hurricane Irma and Maria’s aftermath in Puerto Rico examined their experiences. Participants emphasized significant challenges related to treatment and access to care [35]. Furthermore, the lack of electricity and water, as well as transportation-imposed household limitations, hindered their ability to continue treatment [39].

The HHI is a promising tool for assessing the impact of hurricanes on mental health. However, its psychometric properties and associations with psychological distress among cancer survivors remain understudied. This study aims to (1) investigate the reliability and factor structure of the HHI among cancer survivors and matched controls in Puerto Rico; (2) examine the associations between HHI scores and psychological distress in both groups; and (3) explore the differential effects of hurricane hazards on psychological distress between cancer survivors and controls. By addressing these research questions, this study contributes to a better understanding of the mental health consequences of hurricanes for cancer survivors and informs the development of targeted interventions.

## 2. Methods

### 2.1. Study Design and Participant Recruitment

The present study is a secondary data analysis from a baseline assessment of a non-experimental longitudinal case–control investigation, “Post Hurricane Cancer Care: Patients Needs after Hurricane Maria” [40]. The parent study evaluated the biopsychosocial impact of the hurricane among cancer survivors in Puerto Rico. Most of the recruitment process occurred during community support group meetings and hospital settings between January 2018 and September 2019. A total of 260 participants were recruited; 130 were identified as cancer survivors and 130 controls were matched with the cancer survivors by sex, area code, and age (+ or −5 years). The control group was matched to cancer patients based on gender, age, and area code to minimize confounding factors and ensure comparable groups.

**Figure 1:**
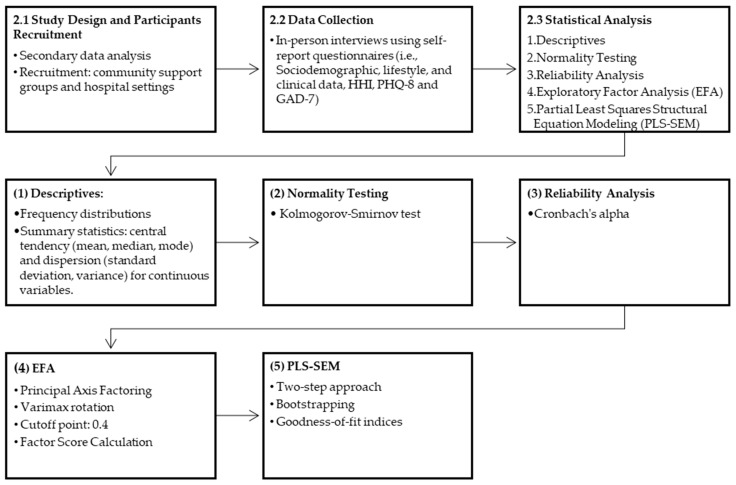
Study methodology.

Trained research assistants invited individuals to participate in the study, and those who agreed to participate provided written informed consent. After recruitment, trained research assistants coordinated an in-person interview to complete the required questionnaires. Participants completed a self-reported pen-to-paper questionnaire in Spanish, supported by doctoral clinical psychology students. The study’s procedures and baseline preliminary results were reported elsewhere [40]. Figure 1 illustrates the research methodology, outlining the study design, data collection procedures, and statistical analysis procedures.

### 2.2. Data Collection

The sociodemographic, lifestyle, and clinical data were collected through a self-report form developed by the researchers of the original study. The questionnaire includes age, marital status, education level, employment status, health, and cancer-related details such as chronic conditions, cancer type, and primary treatment.

The Hurricane Hazards Inventory (HHI), initially named Natural Disaster Outcomes Questionnaire, was developed based on researchers’ personal experiences with Hurricane Maria and a literature review, including the outcomes of natural disasters like hurricanes and earthquakes (see Appendix A) [41,42,43]. Moreover, the research team identified additional outcomes reported by the press, such as a lack of electricity and a collapse of communication systems [44,45]. The inventory aimed to determine the consequences and level of impact suffered by participants in the storm’s aftermath. The questionnaire was reviewed by a research team and was content-validated by individuals who experienced Hurricane Maria and its aftermath. The survey contains 23 Likert-type questions (0 = not at all to 4 = extremely) that explore if and to what degree hurricane-related problems impacted participants during the previous three months (e.g., in the past three months, how much were you bothered by the following: (1) no electricity). The total scores may range from 0 to 92, with higher scores reflecting higher impact. The preliminary reliability results excluding missing values exhibit an excellent internal consistency (α = 0.936) [40].

The Patient Healthcare Questionnaire-8 (PHQ-8) was derived from the Patient Health Questionnaire-9 items (PHQ-9), initially developed by Kroenke and Spitzer and published in 2002 [46]. Currently, it is used in multiple languages, including the Spanish version used in the present study. PHQ-8 is an eight-item self-report measure used to evaluate depression symptomatology and severity (e.g., Over the last two weeks, how often have you been bothered by any of the following problems? (1) Little interest or pleasure in doing things). Each item is rated in frequency on a 4-point scale (0 = not at all, 3 = nearly every day), and total scores may range from 0 to 24. The scores categorize depression symptomatology: 5–9 mild, 10–14 moderate, 15–19 moderate–severe depression, and 20 or more severe depression. In a recent study, the scale obtained an excellent internal consistency of 0.92 with a Puerto Rican sample [47].

The Generalized Anxiety Disorder-7 (GAD-7) was developed by Spitzer, Kroenke, Williams, and Löwe and was published in 2006 [48]. It is used in 73 different languages, including Spanish. The scale allows the identification of probable cases of Generalized Anxiety Disorder (GAD). It assesses symptom severity in anxiety (e.g., Over the last two weeks, how often have you been bothered by the following problems? (1) Feeling nervous, anxious, or on edge). It has ninety-seven items and is evaluated on a scale from 0 to 3, where each symptom is described from not entirely to a few days, more than half the days, or every day. Scores categorize the results as 5–9 moderate, 10–14 possible clinical presence, and 15 or more need treatment for anxiety. The Spanish version was recently validated with a Puerto Rican sample (α = 0.94) [49].

### 2.3. Statistical Analysis

Descriptive Statistics: Frequency distributions and summary statistics (mean, median, mode, standard deviation, and variance) were calculated for continuous variables. The Kolmogorov–Smirnov test was used to assess normality, revealing non-normal distributions for all variables.

Exploratory Factor Analysis (EFA): An Exploratory Factor Analysis (EFA) using Principal Axis Factoring with Oblimin rotation was conducted to determine the factor structure of the Hurricane Hazards Inventory (HHI). The Kaiser–Meyer–Olkin (KMO) measure verified sampling adequacy and Bartlett’s test of sphericity confirmed the suitability of the factor analysis. EFA was chosen due to the lack of previous research on the measure and the absence of clear subscales. A cutoff point of 0.4 was used for pattern matrix correlation. Factor scores were calculated by averaging the items associated with each factor. After accepting a final EFA model, factor scores were then used in subsequent analyses.

Reliability Analysis: Cronbach’s alpha was calculated to assess the reliability of the HHI and its subscales. A cutoff point of 0.4 was used for pattern matrix correlation. Factor scores were calculated by averaging the items associated with each factor [50].

Partial Least Squares Structural Equation Modeling (PLS-SEM): PLS-SEM was conducted using Smart PLS 3.2, following a two-step approach [51]. Bootstrapping was employed to assess the robustness of the results, and goodness-of-fit indices were evaluated. PLS-SEM was selected due to its flexibility in handling non-normal data, its suitability for exploratory research, and its ability to handle complex models with multiple mediators and a moderator. Control variables were not included.

## 3. Results

The study sample consisted of 260 participants who had lived in Puerto Rico during Hurricane Maria, classified into two groups: 130 cancer survivors and 130 matched controls. The majority were women, 78.7%, and the median (interquartile range) age was 58.0 (50.0–66.0) years. The sociodemographic characteristics of the sample are presented in Table 1.

### 3.1. Exploratory Factor Analysis

To explore the factorial structure of the HHI, all 23 items of the instrument were subjected to an Exploratory Factor Analysis (EFA) with oblique rotation (Oblimin with Kaiser Normalization). The Kaiser–Meyer–Olkin measure verified the sampling adequacy for the analysis, KMO = 0.926. Bartlett’s test of sphericity X^2^ (253) = 3797.31, *p* = 0.05, indicated that the correlation structure is adequate for factor analyses. The maximum likelihood factor analysis with a cut-off point of 0.4 and Kaiser’s criterion of eigenvalues greater than 1 yielded the exclusion of six items and a three-factor solution as the best fit, accounting for 62.6% of the variance. The HHI scale with 17 items reflects excellent reliability, with a Cronbach’s alpha coefficient of 0.91. The three factors reflect acceptable to exceptional reliability, ranging from 0.81 to 0.92 across factors. The median for the HHI total score was 11.0 (4.0–26.5) out of 68 possible total scores. See Table 2.

### 3.2. Direct Effects of Hurricane Hazards on Psychological Distress

**Figure 2:**
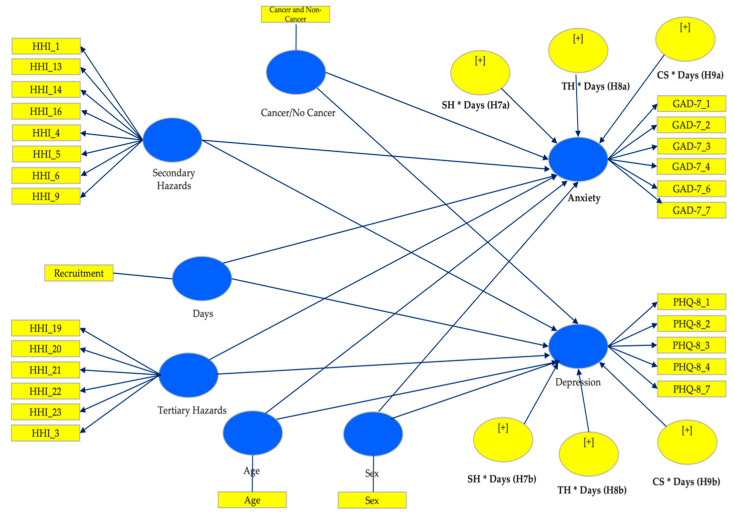
SEM model.

The research model (Figure 2) was analyzed using Smart-PLS 3.2.4, a tool for PLS structural equation modeling (SEM) [51,52,53]. This software evaluates the psychometric properties of the measurement model and estimates the parameters of the structural model.

#### 3.2.1. Measurement Model

The data indicate that the measures are robust regarding their internal consistency and reliability, as evidenced by Cronbach’s alpha and composite reliability. All Cronbach’s alphas and composite reliabilities of the different measures range from 0.823 to 0.937, exceeding the recommended threshold value of 0.70 [53]. Additionally, consistent with the guidelines of Fornell and Larcker [53], the average variance extracted (AVE) for each measure exceeds 0.50, indicating the convergent validity of the measures (see Table 3).

#### 3.2.2. Discriminant Validity Assessment of Constructs Using the Fornell–Larcker Criterion

The discriminant validity of the constructs in the measurement model was assessed using the Fornell–Larcker criterion, as depicted in Table 4. Each construct’s diagonal entry represents the square root of the average variance extracted (AVE), measuring the proportion of variance captured by the construct’s indicators relative to measurement error. For instance, the AVE for “Secondary Hazards” was 0.81 (sqrt = 0.9), indicating strong convergent validity. Evaluating discriminant validity, the correlations between constructs were scrutinized against these AVE values. Notably, correlations between “Secondary Hazards” and “Tertiary Hazards” (0.563), “Anxiety” (0.312), and “Depression” (0.209) were found to be below the corresponding AVE, affirming sufficient distinctiveness between constructs. These findings suggest that each construct sufficiently represents a unique dimension of the underlying phenomena, supporting the robustness of the measurement model in capturing distinct aspects of the variables under investigation.

#### 3.2.3. Discriminant Validity Evaluation Using the Heterotrait-Monotrait Ratio (HTMT)

The heterotrait–monotrait ratio (HTMT) values presented in Table 5 assess the discriminant validity of the constructs in the measurement model. HTMT values below the recommended threshold of 0.85 indicate satisfactory discriminant validity, suggesting that the constructs are sufficiently distinct from each other. For instance, the HTMT ratio for “Secondary Hazards” with “Tertiary Hazards” was 0.64 (95% CI: [0.526, 0.733]), well below the threshold, indicating distinctiveness. Similarly, “Anxiety” and “Depression” exhibited HTMT ratios of 0.54 (95% CI: [0.408, 0.656]) and 0.47 (95% CI: [0.329, 0.605]) with other tertiary hazards, respectively, also meeting the criterion for discriminant validity. Notably, the HTMT ratio for “Depression” with “Anxiety” was 0.89 (95% CI: [0.841, 0.947]), confirming the high reliability and internal consistency of the measurement. These findings underscore the robustness of the measurement model in distinguishing between the latent constructs under investigation.

#### 3.2.4. Results of Structural Model: Explained Variance, Effect Sizes, Predictive Relevance, and Multicollinearity Assessment

Table 6 presents the results of the structural model, highlighting the explained variance (R^2^), adjusted R^2^, effect sizes (f^2^), cross-validated predictive relevance (Q^2^), and variance inflation factors (VIFs) for the latent variables. The model demonstrates that “Tertiary Hazard” explains a substantial amount of variance in both anxiety (R^2^ = 0.281) and depression (R^2^ = 0.229), with an effect size), indicating a significant relationship with anxiety (f^2^ = 0.141) and depression (f^2^ = 0.098). Cross-validated predictive relevance (Q^2^) values indicate robust predictions for both anxiety (Q^2^ = 0.181) and depression (Q^2^ = 0.134). Variance inflation factors (VIFs) are close to or below the threshold of 2, indicating minimal multicollinearity among predictors. These findings underscore the significant impact of “Tertiary Hazard” on anxiety and support the model’s reliability in explaining psychological outcomes within the studied population.

#### 3.2.5. Direct Effects of Predictor Variables on Anxiety and Depression

Table 7 presents the results of the direct effect hypotheses examining the relationships between predictor variables and anxiety (Anx) and depression (Dep). Significant direct effects (*p* < 0.05) were observed for several hypotheses. Specifically, sex (Sex) significantly negatively influenced anxiety (β = −0.121, *p* = 0.019), supporting H1a, but did not significantly affect depression (Dep) (β = −0.089, *p* = 0.134), as per H1b. Cancer status (CS) positively influenced both anxiety (β = 0.198, *p* < 0.001) and depression (β = 0.226, *p* < 0.001), supporting H3a and H3b, respectively. Moreover, “Tertiary Hazard” (TH) showed substantial positive effects on both anxiety (β = 0.405, *p* < 0.001) and depression (β = 0.349, *p* < 0.001), supporting H6a and H6b, respectively. Conversely, age, days, and secondary hazards (SHs) did not show significant direct effects on anxiety or depression. These findings highlight specific demographic and hazard-related factors significantly influencing psychological outcomes, underscoring their importance in understanding anxiety and depression in the studied population.

## 4. Discussion

This study examined the reliability and factor structure of the HHI and its relationship to psychological distress among cancer survivors. It matched controls who were also affected by Hurricane Maria in 2017. A preliminary psychometric analysis of the HHI supported its continued use to assess the consequences and impact experienced by participants in the storm’s aftermath [40].

The Kaiser–Meyer–Olkin value indicated that the sample size was adequate for validating the HHI, even after excluding six items. The psychometric analyses supported a shortened 17-item version of the HHI. Additionally, Bartlett’s test of sphericity showed that the questionnaire demonstrated good construct validity and was suitable for factor analysis. Our results indicated that the 17-item HHI is a valid and reliable measure of hurricane hazards among cancer survivors and matched controls.

The EFA revealed a three-factor structure for the 17-item HHI, with all factors exhibiting acceptable to excellent reliability. In line with the existing literature, two factors could be identified as secondary and tertiary hurricane hazards. Primary hazards refer to direct impacts like high winds, flooding, rain, and heavy waves. Secondary hazards, included in the HHI, are related to the loss of essential resources such as electricity, water, and food. Tertiary hazards, also included in the HHI, represent the indirect consequences of secondary hazards, including emotional distress, family separation, and security concerns [19,20]. The third factor, focused on electric generator expenses, was excluded from further analysis due to a lack of theoretical foundation. A validated measure of psychological distress after Hurricane Maria, the Post-Hurricane Distress Scale (PHDS), was used. While the PHDS assesses trauma and distress, it does not specifically evaluate the impact of material and social losses associated with secondary and tertiary hazards.

Hurricane hazards have been well documented as factors in the etiology of depression and anxiety [24,54,55]. Our results showed that hurricane hazards impact the mental health of individuals, primarily cancer survivors. Preliminary studies on our sample reported that the time that had passed after the hurricane was a factor related to the impact of the disaster on mental health [40]. However, our results suggested that the loss of social support (tertiary) directly impacts the mental health of those who experience a natural disaster. Likewise, being a man seems to be associated with presenting anxiety symptoms and being a cancer patient with depressive symptoms. This supports previous findings where natural disaster psychological impact was reported, particularly the loss of social support and resources. The adverse effect of natural disasters on the psychological functioning of survivors was maintained in the years after the events [56]. Individuals with a low income, low social support, and high levels of non-organizational religiosity presented the worst symptomatology [27,57].

The findings of this study make a significant contribution to the literature on the impact of natural disasters on vulnerable populations. These results can be interpreted through the lens of syndemic theory [58,59,60,61]. Syndemic theory examines the detrimental interactions between diseases and social conditions, emphasizing the mechanisms of these interactions. Our research illustrates the negative interaction between a cancer diagnosis and the experience of a natural disaster, such as a hurricane, and the subsequent impact on depression and anxiety symptoms in our sample.

The syndemic framework provides a conceptual model that highlights the interconnectedness of multiple epidemics within specific contexts. In natural disasters like hurricanes, the syndemic framework elucidates the complex interplay between hurricane hazards and non-communicable diseases, such as cancer. This interplay can lead to significant changes in disease management, including access to healthcare services and medication. Applying the syndemic framework to our research in Puerto Rico, an area frequently affected by hurricanes and other hazards, involves thoroughly examining how various social determinants of health, infectious diseases, and non-communicable diseases interact within vulnerable populations. By identifying the specific social and medical factors and the social and structural inequities that contribute to disease clustering and adverse health outcomes, we can better address barriers to effective healthcare delivery.

The lasting impact of Hurricane Maria, combined with Puerto Rico’s location in an active hurricane zone, provides valuable insights into managing hazards and emergencies. Puerto Rico’s economy faced significant disruption, revealing broader economic vulnerabilities common to hurricane-affected regions. Disasters often lead to prolonged economic declines, as seen in Puerto Rico and Florida, where employment recovery can take years [62,63]. The delayed implementation of mitigation strategies, such as flood defenses and stricter building codes, worsened the damage, highlighting the importance of timely adaptation. Financial resilience and swift recovery measures are crucial for mitigating long-term economic impacts [64]. Additionally, healthcare and academic institutions played a vital role in maintaining critical services, despite the complexity of managing healthcare infrastructure post-hurricane. Ensuring these systems remain adaptable is essential for both immediate relief and long-term recovery [65]. Improved public communication strategies, such as clearer evacuation messaging, are also key to reducing casualties and fostering faster recovery during future storms [66]. The interplay between natural disasters like hurricanes and chronic conditions such as cancer underscores the complexity of managing healthcare in such contexts. It requires an approach that accounts for the interdependencies between environmental hazards and chronic diseases. This perspective leads to more effective interventions and policies to improve health outcomes for individuals facing multiple interacting health challenges. Viewed through the syndemic framework, our findings highlight the need for integrated health strategies that address the compounded effects of disease and social adversity following natural disasters on vulnerable populations.

## 5. Conclusions

Our study highlights the ongoing impact of natural disasters on vulnerable populations, particularly cancer patients, by validating a reliable measure of natural disaster hazards. We found that the mental health effects of these events persist over time, exacerbated by the loss of social support. This has significant practical implications for post-disaster interventions, allowing healthcare providers to better prioritize resources and psychosocial support based on the specific secondary and tertiary hazards experienced by individuals. Clinicians and policymakers can use these insights to design more holistic interventions that treat psychological distress and consider social and material losses, which are crucial in disaster recovery efforts. This underscores the need for strong community networks and public policies aimed at prevention and new challenges. An interdisciplinary approach integrating medical, psychological, and social services is essential for supporting cancer patients before, during, and after disasters.

The present results contributed to developing preventive mental healthcare for cancer patients and survivors, especially Hispanic breast cancer survivors in southern Puerto Rico (Protocol#: 2303136895R001). These patients face social and structural inequalities, including limited access to psychosocial and psycho-oncology mental healthcare (PO-MHC). Despite Institute of Medicine (IOM) recommendations for such services, our study found limited social-supportive services and a shortage of PO-MHC services in southern Puerto Rico, likely due to a lack of specialists and geographical disparities.

Addressing these barriers requires solving structural issues and tackling mental health stigma among Hispanics. This study aims to adapt a culturally sensitive community toolbox for community health workers to address social determinants of mental health. This approach integrates community efforts with academic resources to address service shortages, stigma, and psychological distress. Our findings, framed through syndemic theory, highlight the compounded effects of chronic psychological distress, environmental hazards, and social inequities on health outcomes. This multi-level intervention empowers vulnerable communities and offers a transformative population-level impact, serving as a model for addressing the multifaceted challenges faced by cancer patients and survivors in underserved regions.

## 6. Limitations

Some of this study’s limitations are its small sample size and the selection’s reliance on availability, which hamper generalizability. Additionally, the statistical analysis was not controlled by relevant variables such as type and stage of cancer and sociodemographic variables such as employment status. The sample was also relatively homogeneous, and future psychometric analyses with greater and more diverse samples are essential to address these limitations. Future research should address whether hurricane hazards’ impact on psychological distress is consistent over time.

While our study matched controls with cancer survivors based on sex, area code, and age, we did not assess their underlying health conditions. This is a limitation of our study, as the presence of other health conditions, such as heart disease and diabetes, could potentially influence individuals’ perceptions of hurricane hazards and their psychological responses to disasters. Future studies should consider assessing these factors to provide a more comprehensive understanding of the relationship between hurricane hazards and psychological distress, particularly in vulnerable populations like cancer survivors.

Furthermore, future research should validate the psychometric properties of the HHI in other vulnerable populations, evaluate its consistency over time, and increase their sample size to enhance the generalizability of findings. Conducting a multigroup analysis to examine the HHI’s performance across different subgroups can also provide valuable insights.

Additionally, investigating protective factors, such as resilience and social support, can help identify factors that may mitigate the negative impact of hurricane hazards on psychological well-being. Developing interventions and policies that address the tertiary hazards of disasters, such as loss of social support, and prioritizing mental health services, particularly for vulnerable populations like cancer survivors, is crucial for improving the overall well-being of affected communities. Preparedness and community empowerment can also play a vital role in enhancing the resilience of individuals and communities facing disasters.

## Figures and Tables

**Table 1:** Sociodemographic characteristics and cancer diagnosis of participants (*n* = 260).

Variables	f (%), $\overset{-}{x}$
Gender Female Male	199 (78.7) 54 (21.3)
Age, median (interquartile range) Education Level * Less than High School* * High School or higher* Marital Status * Married/Consensual Union* * Not Married* Employment Status * Employed* * Unemployed* * Retired/Disabled* * Other* Cancer Diagnosis * Yes* * No*	58.0 (50.0–66.0) 21 (9.3) 197 (90.7) 125 (55.8) 99 (44.2) 61 (23.5) 43 (16.5) 100 (38.5) 17 (6.5) 130 (50.0) 130 (50.0)

**Table 2:** Hurricane Hazards Inventory pattern matrix.

Items	Factor Loadings		
	1	2	3
1. No electricity	0.802		
2. No water	0.842		
3. Lack of food	0.707		
4. Lack of food security	0.814		
5. Difficulty accessing roads	0.682		
6. Long lines at gas stations	0.788		
7. Traffic jam	0.620		
8. Difficulties with communication	0.787		
9. Loss of a loved one		0.475	
10. Employment lay-off or reduction in labor hours		0.497	
11. Lack of personal space		0.394	
12. Lack of social support		0.812	
13. Family separation		0.755	
14. Loss of vehicle		0.634	
15. Loss of security for children and family		0.608	
16. Cost of generator maintenance (gas or diesel, oil)			0.707
17. Cost of generators acquisition			0.658

**Table 3:** Correlation matrix of latent variables, average variance extracted (AVE), Cronbach’s alpha (α), and composite reliability (CR).

Scale	Item	Outer Loading	AVE	Cronbach’s Alpha	Composite Reliability
Secondary Hazards	HHI_1	0.753	0.651	0.924	0.937
	HHI_4	0.847			
	HHI_5	0.818			
	HHI_6	0.844			
	HHI_9	0.811			
	HHI_13	0.810			
	HHI_14	0.775			
	HHI_16	0.790			
Tertiary Hazards	HHI_3	0.589	0.537	0.823	0.873
	HHI_19	0.659			
	HHI_20	0.846			
	HHI_21	0.803			
	HHI_22	0.686			
	HHI_23	0.782			
Anxiety	GAD-7_1	0.854	0.694	0.912	0.932
	GAD-7_2	0.846			
	GAD-7_3	0.829			
	GAD-7_4	0.856			
	GAD-7_5	0.815			
	GAD-7_7	0.797			
Depression	PHQ-8_1	0.824	0.641	0.859	0.899
	PHQ-8_2	0.868			
	PHQ-8_3	0.740			
	PHQ-8_4	0.818			
	PHQ-8_7	0.743			

**Table 4:** Fornell–Larcker criterion.

	Scale	1	2	3	4
1.	Secondary Hazards	(0.81)			
2.	Tertiary Hazards	0.563	(0.73)		
3.	Anxiety	0.312	0.471	(0.83)	
4.	Depression	0.209	0.397	0.209	(0.80)

**Table 5:** Heterotrait-monotrait ratio (HTMT).

	Scale	1	2	3	4
1	Secondary Hazards				
2	Tertiary Hazards	0.64 [0.526; 0.733]			
3	Anxiety	0.33 [0.193; 0.452]	0.54 [0.408; 0.656]		
4	Depression	0.21 [0.124; 0.356]	0.47 [0.329; 0.605]	0.89 [0.841; 0.947]	

**Table 6:** Structural model result.

Latent Variable	R^2^	R^2^ Adj.	Effect Size (f^2^)		Q^2^	VIF
			Anx	Dep		
Sex			0.019	0.010		1.042
Age			0.003	0.012		1.095
Cancer Status			0.050	0.060		1.098
Secondary Hazard			0.009	0.002		1.203
Tertiary Hazard			0.141	0.098		1.617
SH * Days			0.000	0.001		1.675
TH * Days			0.000	0.001		1.664
CS * Days			0.002	0.009		1.042
Anxiety	0.281	0.254			0.181	
Depression	0.229	0.201			0.134	

**Table 7:** Results of direct effect hypotheses.

Hypothesis		Beta	SE	t-Value	*p*-Value	CIBC		Decision
						2.5%	97.5%	
**H_1a_:**	Sex→Anx	−0.121 *	0.051	2.346	**0.019**	−0.220	−0.017	Supported
**H_1b_:**	Sex→Dep	−0.089	0.059	1.499	0.134	−0.200	0.032	Not Supported
**H_2a_:**	Age→Anx	−0.05	0.057	0.873	0.383	−0.162	0.059	Not Supported
**H_2b_:**	Age→Dep	−0.100	0.057	1.756	0.079	−0.212	0.010	Not Supported
**H_3a_:**	CS→Anx	0.198 *	0.057	3.485	**<0.001**	0.086	0.310	Supported
**H_3b_:**	CS→Dep	0.226 *	0.059	3.86	**<0.001**	0.110	0.335	Supported
**H_4a_:**	Days→Anx	0.021	0.056	0.378	0.706	−0.090	0.126	Not Supported
**H_4b_:**	Days→Dep	0.074	0.060	1.239	0.215	−0.047	0.188	Not Supported
**H_5a_:**	SH→Anx	0.103	0.076	1.35	0.177	−0.048	0.251	Not Supported
**H_5b_:**	SH→Dep	0.052	0.084	0.619	0.536	−0.114	0.213	Not Supported
**H_6a_:**	TH→Anx	0.405 *	0.079	5.139	**<0.001**	0.244	0.554	Supported
**H_6b_:**	TH→Dep	0.349 *	0.083	4.191	**<0.001**	0.173	0.501	Supported

Note: * Significant.

## Data Availability

Data can be provided upon request.

## Appendix A. Hurricane Hazards Inventory

The Hurricane Hazards Inventory

**Instructions:** Below is a list of situations that people may suffer in response to a natural disaster. Please read each situation carefully and then circle one of the numbers to the right to indicate how much you have been bothered by that problem in **the past three months**.

**In the Past Three Months, How Much Were You Bothered by**	**Not** **at All**	**A Little Bit**	**Moderately**	**Quite a Bit**	**Extremely**
1. No electricity	0	1	2	3	4
2. No water	0	1	2	3	4
3. Lack of food	0	1	2	3	4
4. Lack of food security	0	1	2	3	4
5. Difficulty accessing roads	0	1	2	3	4
6. Long lines at gas stations	0	1	2	3	4
7. Traffic jam	0	1	2	3	4
8. Difficulties in communications	0	1	2	3	4
9. Loss of a loved one	0	1	2	3	4
10. Employment lay-off or reduction in labor hours	0	1	2	3	4
11. Lack of personal space	0	1	2	3	4
12. Lack of social support	0	1	2	3	4
13. Family separation	0	1	2	3	4
14. Loss of vehicle	0	1	2	3	4
15. Loss of security for my children and family	0	1	2	3	4
16. Cost of generator maintenance (gas or diesel, oil)	0	1	2	3	4
17. Cost of generators acquisition	0	1	2	3	4
